# Clinical and pathological challenges in the diagnosis of late-onset biliary atresia: four case studies

**DOI:** 10.1590/1414-431X20154808

**Published:** 2016-02-02

**Authors:** J.P.U. Fontenele, A.A. Schenka, G. Hessel, V.M. Jarry, C.A.F. Escanhoela

**Affiliations:** 1 Departamento de Patologia, Faculdade de Ciências Médicas, Universidade Estadual de Campinas, Campinas, SP, Brasil; 2 Departamento de Farmacologia, Faculdade de Ciências Médicas, Universidade Estadual de Campinas, Campinas, SP, Brasil; 3 Departamento de Pediatria, Faculdade de Ciências Médicas, Universidade Estadual de Campinas, Campinas, SP, Brasil

**Keywords:** Kasai operation, Cholestatic syndrome, Infants

## Abstract

Biliary atresia (BA) is classically described at the neonatal age. However, rare cases of BA in older infants have also been reported. We report four cases of late-onset BA in infants older than 4 weeks (3 males, 1 female), and describe the diagnostic and management difficulties. One of the cases had a late-onset (29 weeks) presentation with a successful surgical procedure. We highlight the importance of this unusual differential diagnosis in infants with cholestatic syndrome, who may benefit from Kasai surgery, regardless of age.

## Introduction

Biliary atresia (BA) is still a challenge for clinicians and researchers. This disease is classically described in neonates and its early diagnosis is fundamental for a better prognosis (1). Viral etiological agents, such as cytomegalovirus, defects in morphogenesis of the biliary tree, and genetic, epigenetic, ischemic, inflammatory, immune, and even toxic factors (2,3), alone or combined, have been implicated in the etiopathogenesis of BA. However, the mechanisms of BA are still largely unknown. BA commonly manifests in the first month of life. Nevertheless, late-onset forms, as well as successful Kasai portoenterostomy procedures in older children, have been observed (4), reflecting the heterogeneous nature of BA. Yang et al. (5) classified BA according to the time of onset of symptoms as early-onset and late-onset forms. They defined these early- and late-onset forms as cholestatic jaundice and fecal hypocholia before and after 2 weeks of life, respectively.

In this report, we describe four cases of late-onset BA in infants older than 4 weeks and their histopathological findings. We highlight the importance of this diagnosis in infants with cholestatic syndrome and the diagnostic and management difficulties. Our findings reinforce that failure of early diagnosis may result in delayed indication of optimal surgical procedures.

## Case Reports

All cases of BA originated from patients who were treated between 2006 and 2012 at the Departamento de Pediatria, Faculdade de Ciências Médicas, Universidade Estadual de Campinas (FCM, UNICAMP), Campinas, SP, Brazil. The present report was approved by the Ethics Committee of the Departamento de Pediatria, FCM, UNICAMP, Brazil. After chart analyses and case descriptions, histological slides were reviewed independently by two pathologists.

### Case 1

This patient was a male infant, aged 31 weeks and 1 day, with a history of jaundice, choluria, and fecal hypocholia that had begun 2 weeks prior. The infant was born at 40 weeks of gestational age, weighed 3600 g, and had no history of neonatal jaundice or other comorbid conditions. The mother had gestational diabetes and was exclusively treated with diet. Baseline biochemical tests were consistent with cholestatic syndrome (baseline biochemical tests of all four cases are shown in Table 1). Serology for cytomegalovirus was negative. Abdominal ultrasound at admission to the hospital showed an enlarged liver, with slightly heterogeneous parenchyma, a distended gallbladder, and dilatation of the intrahepatic and extrahepatic systems. After an inconclusive clinical investigation, the infant underwent exploratory laparotomy at 37 weeks and 1 day old. We observed a gallbladder with a thin and distended wall, adherence of the intestinal loops to the hilum of the liver, and an indurated, fibrotic, extrahepatic bile duct system. Transoperative cholangiography showed that there was failure to fill at the junction of the hepatic ducts and a lack of drainage to the small intestine. Kasai portoenterostomy was the treatment of choice. The infant showed significant clinical improvement and normalization of bilirubin levels 1 week after surgery. He is currently asymptomatic after 65 months of follow-up.

**Table:**
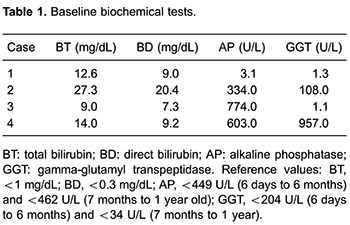

### Case 2

This patient was a male infant, aged 17 weeks and 4 days, with a history of isolated jaundice since 8 weeks of life. There were no complications in prenatal care. The infant was born at 40 weeks and 2 days of gestational age, and weighed 3150 g. Baseline clinical investigations showed an isolated increase in direct bilirubin serum levels, a liver with a heterogeneous parenchyma, and a non-contractile gallbladder on abdominal ultrasound. Serology for cytomegalovirus was negative. Percutaneous liver biopsy was performed, with histopathological findings consistent with BA, at a stage of focal nodular transformation. Because of the older age at onset and the atypical clinical picture for BA, primary sclerosing cholangitis was initially considered. Final diagnosis was dependent on findings at exploratory laparotomy, which showed a macroscopically normal gallbladder, an extrahepatic biliary system, as well as the usual appearance of the liver. Transoperative cholangiography revealed a minute streak of contrast material ascending the intrahepatic bile duct system. We decided to interrupt the Kasai operation and only perform cholecystectomy and wedge biopsy of the liver. The child rapidly progressed to liver cirrhosis and the diagnosis of biliary atresia was considered at this moment. The child deteriorated before transplantation was attempted.

### Case 3

This patient was a male infant, aged 14 weeks, with clinical presentation of jaundice, choluria, and fecal hypocholia for 2 weeks. The infant was born at 39 weeks gestational age and weighed 3320 g. He presented with jaundice on the second day of life, was treated with phototherapy, and discharged from the hospital with improvement of symptoms. The mother had a history of deep venous thrombosis and used prophylactic heparin during pregnancy. On initial investigation, biochemical tests were typical of cholestatic syndrome. Serology for cytomegalovirus was negative. Abdominal ultrasound failed to visualize the gallbladder during fasting. Percutaneous liver biopsy was attempted without success. Our treatment of choice was exploratory laparotomy at 15 weeks and 4 days of age. This procedure showed the gallbladder and extrahepatic bile duct systems with total atresia. Kasai portoenterostomy was performed. After surgery, there was partial improvement of clinical and laboratory parameters, and the infant received referral for liver transplantation, which was performed at another hospital. He was lost to follow-up immediately after the procedure.

### Case 4

This patient was a female infant, aged 9 weeks and 4 days, with clinical presentation of jaundice, choluria, and fecal hypocholia, with an onset of 5 weeks. Prenatal care showed no complications. She was born at a gestational age of 39 weeks, weighed 2625 g, and had no comorbid conditions. Baseline biochemical tests were consistent with cholestatic syndrome. Serology for cytomegalovirus was negative. Abdominal ultrasound revealed a hypoplastic gallbladder and a liver with a normal aspect. Percutaneous liver biopsy showed histopathological findings typical of BA. Exploratory laparotomy at 10 weeks and 5 days old showed atresia of the extrahepatic bile duct system, an intensely hypoplastic gallbladder, and an enlarged, firm, micronodular liver. After surgery, the infant progressed with partial improvement in clinical and laboratory parameters. She was referred for liver transplantation, which was performed at another hospital, and was lost to follow-up afterward.

## Results

**Figure 1:**
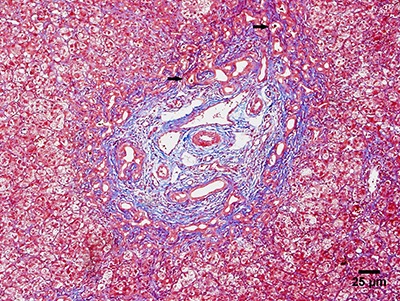
Photomicrograph showing the main histological findings of biliary atresia A portal space with ductular proliferation and biliary plugs (arrows) can be seen. Masson trichrome stain, ×100.

**Figure 2:**
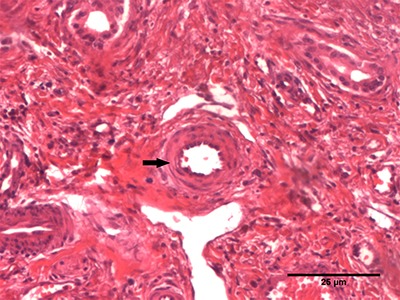
Photomicrograph showing thickening of the arterial medial layer (arrow). Hematoxylin and eosin stain, ×200.

All liver biopsies that were obtained during exploratory laparotomy exhibited a morphological pattern typical of large bile duct obstruction, with severe portal biliary reaction, ductal, intracanalicular, and hepatocytic cholestasis, and cholatestasis. We also observed focal nodular regeneration and giant cell transformation of some sparse hepatocytes (Figure 1). Thickening of the portal branches of the hepatic artery was also observed (Figure 2). Case 1 exhibited type 2 porta hepatis (6), a common bile duct with ductal lumens in the midst of loose fibrous tissue, and a gallbladder with considerable luminal dilatation. Case 3 also demonstrated type 2 porta hepatis, associated with chronic inflammation, with an extrahepatic biliary system and a gallbladder with complete atresia. In case 4, biliary remnants were not visualized, raising the question as to whether it was type 1 porta hepatis or an artifact. The gallbladder showed the usual histological aspect as in cases 2 and 4.

## Discussion

Since 1959, Kasai portoenterostomy has been the only treatment, with the exception of liver transplantation, that effectively changes the prognosis of patients with BA. Several studies have sought objective clinical, histopathological, laboratory, or imaging criteria that could help in treatment decision-making and have prognostic value in children with BA. In this context, whether there is an age limit for the Kasai procedure is unknown. Large series have associated the success of this surgery to the age of the patients; the younger the patient, the better the prognosis. However, because BA has a higher incidence in neonates, these results might have been biased against analysis of age alone. This is probably because in several cases undergoing late surgery, diagnosis had been made at an advanced stage of disease. BA is currently accepted as a heterogeneous disease, with various forms of clinical presentation. Therefore, the distinction between “late diagnosis” and “late onset” of BA may be important. Late-onset forms with a shorter duration of disease are likely to have a favorable outcome after the Kasai procedure, even when the patient is older. Nevertheless, there are also case reports with a late diagnosis where the patient benefitted from the Kasai operation (7).

Our four cases did not have the same outcome. The first case was unusual because the child was 31 weeks old and ultrasound at admission showed intra- and extrahepatic bile duct dilatation. This finding is only observed in approximately 5% of biliary atresia patients with obstruction at the common bile duct (8). The initial differential diagnosis did not include BA. To the best of our knowledge, there are no similar reports of onset of BA later than 29 weeks of life. Diagnosis was made by transoperative cholangiography and supported by histopathological findings. Even at an older age, the Kasai procedure was the treatment of choice. The child had a favorable clinical course and continues to be asymptomatic until the present date. Clinical features of case 1 suggest an acquired form of BA, which can be associated with perforation of biliary tracts, as described by Davenport et al. (9). However, there were no signs of bile duct perforation or a history of surgical manipulation. The morphological findings were characteristic of isolated biliary atresia.

In contrast, in case 2, the infant did not undergo Kasai surgery, even with previous histopathological findings suggestive of BA. The clinical diagnosis was primary sclerosing cholangitis because of the atypical clinical picture of jaundice at 8 weeks of life, low serum gamma-glutamyl transpeptidase levels, and the absence of fecal hypocholia. The final diagnosis depended exclusively on surgical findings and cholangiography that was performed during exploratory laparotomy. In this case, transoperative cholangiography showed a minute streak of contrast medium ascending the intrahepatic bile duct system. This finding was interpreted by the surgeon as information that ruled out the diagnosis of BA. This infant progressed with liver cirrhosis and its complications, and missed the opportunity for an effective Kasai procedure. The image shown by cholangiography most probably represented the evolutionary stage of the disease, with partial obstruction of the bile duct system. This case highlights the difficulties in diagnosing BA, also demonstrating variability in transoperative cholangiography findings, which are considered the gold standard for diagnosis. The third and fourth cases, even at older ages and with approximately 4 and 6 weeks of symptoms, respectively, underwent Kasai portoenterostomy. However, the outcome was unfavorable and the infants were referred to the surgical service for liver transplantation.

Common morphological alterations of BA in liver biopsy specimens include a periportal ductular reaction, cholestasis, and portal expansion with edematous fibroplasia, in addition to fibrous septa and nodules in advanced stages. Findings such as ductal plate malformation have also been observed in some cases, suggesting an intrauterine insult in the physiopathogenesis of BA, but with no prognostic significance. Some researchers have described thickening of the median layer of the portal branches of the hepatic artery as part of the histopathological criteria of BA (10,11). A morphometric and immunohistochemical study on angiogenic factors (vascular endothelial growth factor A) also supported this observation, suggesting that ischemia/hypoxia phenomena participate in the etiopathogenesis of BA (12). An angiographic study in patients with BA showed alterations in branches of the hepatic artery, such as tortuosities, formation of vascular arterial tuft-like lesions, and occlusion of peripheral arterial branches, suggesting that these findings may be characteristic of BA (13). Furthermore, an increase in the resistance index of the hepatic artery, measured by Doppler ultrasound, has been associated with a worse prognosis in patients with BA (14).

Classification of BA has experienced several modifications throughout the years and is inconsistent among researchers. Two clinical presentations of BA have been classically described (2). One presentation is embryonic BA, characterized by persistent jaundice at birth, associated with non-hepatic congenital structural alterations, also called the early, fetal, or syndromic form. The other presentation is perinatal BA, with a jaundice-free interval at birth, is present alone, and is also called the late or non-syndromic form. Davenport et al. proposed a new term of “biliary atresia splenic malformation syndrome” and defined clinical criteria for the forms of embryonic BA (15). New clinical forms, supported by imaging and laboratory tests, and prognostic characteristics have emerged, such as a cystic BA and a form associated with cytomegalovirus infection (2). An acquired form of BA, secondary to perforation of the extrahepatic biliary system or previous surgical manipulation, has been described with the same morphological findings (9). The question of why the acquired form of BA does not develop in older children, or in adults, remains unanswered. Variability of frequently superimposed clinical presentations that are difficult to classify reinforces the hypothesis that BA represents a group of disorders with distinct etiopathogenic mechanisms. All of these disorders have the final pathway of progressive destructive inflammatory obliterative cholangiopathy in common.

In summary, we describe four late-onset cases of BA, highlighting the importance of this differential diagnosis not only in neonates, but also in infants with cholestatic syndrome. Every case should be evaluated, based on clinical, laboratory, histopathological, surgical, and imaging criteria, which are highly variable. BA is still a disease with many unanswered questions, and its diagnosis can be a challenge. Therefore, the experience of the reference center is extremely important. Management should be individualized, regardless of the age of the patient. We are currently performing a morphometric study of the portal branches of the hepatic artery in all cases with wedged liver biopsy in our service. We hope to understand the actual associations between these morphologic data and BA, its clinical forms, and the time of disease.
